# Human Papillomavirus (HPV)-Associated Cancers Among Hispanic Males in the United States: Late-Stage Diagnosis by Country of Origin

**DOI:** 10.1177/10732748231218088

**Published:** 2023-11-28

**Authors:** Seiichi Villalona, Satsuki Villalona, Daisy Reinoso, Simone Sukhdeo, Antoinette M. Stroup, Jeanne M. Ferrante

**Affiliations:** 1 Department of Medicine, University of Pennsylvania Perelman School of Medicine, Philadelphia, PA, USA; 2 21798 Hospital of the University of Pennsylvania, Philadelphia, PA, USA; 3 3280 The College of New Jersey, Ewing, NJ, USA; 4 12287 Rutgers Robert Wood Johnson Medical School, Piscataway, NJ, USA; 5 Department of Biostatistics and Epidemiology, 51893 Rutgers School of Public Health, Piscataway, NJ, USA; 6 Rutgers Cancer Institute of New Jersey, New Brunswick, NJ, USA; 7 Department of Family Medicine and Community Health, 12287 Rutgers Robert Wood Johnson Medical School, New Brunswick, NJ, USA

**Keywords:** human papillomavirus, Hispanic, males, late-stage cancer

## Abstract

**Introduction:**

The epidemiology of human papillomavirus (HPV)-associated cancers has changed since the development of the multivalent vaccine. This is evidenced by the decline in incidence of cervical cancers in the post-vaccine era. By contrast, studies have reported the rise in incidence of these cancers in males. Though little is known regarding HPV-associated cancers in males, Hispanic males have been largely excluded from research on these cancers.

**Objective:**

The purpose of this study was to examine the differences in late-stage diagnosis of HPV-associated cancers (oropharyngeal, anorectal, or penile) among subgroups of Hispanic males in the U.S.

**Methods:**

We performed a population-based retrospective cohort study using the 2005–2016 North American Association of Central Cancer Registries Cancer in North America Deluxe data file (n = 9242). Multivariable logistic regression modeling was used in studying late-stage diagnosis.

**Results:**

There were no differences in late-stage diagnosis of oropharyngeal cancer between Hispanic subgroups. Higher odds of late-stage penile cancers were observed among Mexican and Puerto Rican males relative to European Spanish males. Lower odds of late-stage anorectal cancers were observed among Central or South American and Puerto Rican males. Having Medicaid or no insurance were associated with late-stage diagnosis for all cancers.

**Conclusion:**

Certain subgroups of Hispanic males have higher odds of late-stage HPV-associated cancer diagnosis based on country of origin and insurance status. These findings call for improved efforts to increase HPV vaccination, particularly among these subgroups of Hispanic males. Efforts to improve health care access and early detection from health care providers are also needed.

## Introduction

In the United States, human papillomavirus (HPV) is the most common sexually transmitted infection (STI) and is purported to cause an estimated 19,925 cases of HPV-associated cancers among males annually.^1^ Most of the literature on HPV-associated cancers have focused on females and the virus’ link to cervical cancers, despite it being associated with 72% of squamous cell carcinomas (SCCs) of the oropharynx, 91% of anorectal SCCs, and 63% of penile SCCs.^2^ HPV-associated cancers are preventable, and vaccination remains the best strategy in mitigating adverse health outcomes since there are currently no routine screening recommendations for HPV-associated cancers among males.^3^ Furthermore, despite studies demonstrating that males have a 3-fold higher prevalence of HPV positivity and are less likely to clear HPV infections,^4^ HPV vaccination campaigns have primarily targeted females.^5^ Although the Advisory Committee on Immunization Practices (ACIP) recommended the HPV vaccine as protection for females in 2006, the recommendation did not extend to males until 2011. Since October 2011, ACIP recommends the HPV vaccine to 11- to 12-year-old males, with 9- to 21-year-old males also considered age-eligible.^6^ More recently, the HPV 9-valent vaccine (Gardasil 9, Merck) was approved by the FDA for both men and women through 45 years of age in preventing HPV associated malignancies affecting the oropharynx, cervix, anorectal tract, and penis.^7^

Although studies have suggested that HPV vaccine initiation has increased among males, completion rates continue to lag behind females.^8^ A study based on the 2013 National Immunization Survey Teen (NIS-Teen) found that 34.6% of boys between the ages of 13–17 had one dose of the HPV vaccine while series completion (≥3 doses) was only 13.9%.^9^ Completion rates were significantly higher among Hispanics (20.3%) compared to Non-Hispanic (NH) black (15.7%) and white males (11.1%).^10^ Despite this improvement, Hispanic adolescent males continue to have lower rates of HPV vaccination initiation and completion than Hispanic females (21% vs 39% respectively).^2,11^

Several studies have examined factors associated to HPV vaccination uptake and knowledge about HPV among Hispanic males.^2,11,12^ However, there have been very few studies that include Hispanics and/or males when examining health outcomes of HPV-associated cancers. For instance, Hispanic males have the highest age-adjusted incidence rate of HPV-associated penile cancer compared to non-Hispanic males.^13^ In addition, Hispanic males are over 4 times more likely to develop HPV-associated oropharyngeal cancer than Hispanic females,^1^ with lower socioeconomic status and education being major risk factors.^14^ This is particularly concerning given that Hispanics have higher HPV-associated oropharyngeal cancer mortality compared to NH Whites,^15^ which is further reflected when stratifying by lower socioeconomic status.^16^ Most of the existent literature on HPV-associated cancers has predominantly focused on understanding NH White-Black disparities.^17-19^ Furthermore, few studies pertaining to HPV-associated cancers have included Hispanics/Latinos as distinct groups, particularly among males. If Hispanics were included, researchers focused only on a certain state, one type of cancer, or Medicare beneficiaries.^20,21^ Given the heterogeneity within the broader category of Hispanics, it is important to analyze the data among the different subgroups based on country of origin, such as European Spanish, Cuban, Mexican, Dominican, Central/South American, and Puerto Rican.^22^ In doing so, the preventative and treatment efforts can be better targeted within this diverse population and in geographic regions where larger numbers of Hispanic males reside. To the best of our knowledge, this is the first study to examine differences in late-stage diagnosis of HPV-associated cancers (i.e., oropharyngeal, penile, and anorectal) among Hispanic males by country of origin.

## Methods

### Study Design and Sample

This is a population-based retrospective cohort study of Hispanic males in the U.S. diagnosed with invasive (i.e., not including in situ) oropharyngeal, anorectal, or penile cancers from 01/01/2005-12/31/2016 in the North American Association of Central Cancer Registries (NAACCR) Cancer in North America (CiNA) Deluxe data file. NAACCR CiNA is the most comprehensive cancer incidence database, given its coverage of approximately 93% of the population living in the U.S. This database contains de-identified demographic, cancer type, and treatment information from population-based cancer registries across the U.S. and Canada. At the time the study was conducted (2019–2020), the full data file from NAACCR was available through 2016. We included 12 years of data to have a sufficient number of cases for each HPV-associated cancer in Hispanic males. The Surveillance, Epidemiology, and End Results (SEER)*Stat software program was used to access the data, which was then exported into the Statistical Package for the Social Sciences (SPSS) version 27 (IBM, Armonk, New York) for advanced analyses. This study was approved and determined exempt from review by the Institutional Review Boards at NAACCR and Rutgers University.

**Figure 1.**
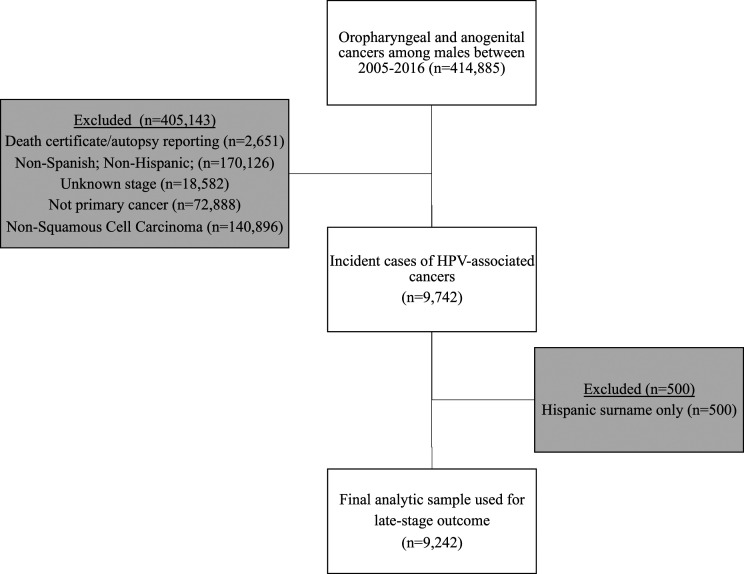
Study cohort derivation.

Cases identified in the NAACCR CiNA file were downloaded onto a Microsoft Excel document, where data cleaning was performed based on inclusion and exclusion criteria. We included Hispanic males with known tumors in anatomic sites under the International Classification of Diseases for Oncology (ICD-O)-3. Cases were considered to be oropharyngeal cancers if they were located at the base of tongue (C01.9); lingual tonsil (C02.4); overlapping lesion of tongue (C02.8); soft palate (C05.1); uvula (C05.2); tonsillar fossa (C09.0); tonsillar pillar (C09.1); overlapping lesion of tonsil (C09.8); tonsil (C09.9); vallecula (C10.0); anterior surface of epiglottis (C10.1); lateral wall of oropharynx (C10.2); posterior wall of oropharynx (C10.3); branchial cleft (C10.4); overlapping lesion of oropharynx (C10.8); oropharynx (C10.9); pharynx (C14.0); Waldeyer’s ring (C14.2); overlapping lesion of lip, oral cavity, and pharynx (C14.8). The anorectal cancer category was determined by malignancies in the rectum (C20.9); anus (C21.0); anal canal (C21.1); cloacogenic zone (C21.2); overlapping lesion of rectum, anus, and anal canal (C21.8). Penile cancers were classified by those found at the prepuce (C60.0); glans penis (C60.1); body of penis (C60.2); overlapping lesion of penis (C60.8); and penis (C60.9). Figure 1 outlines how the final study sample of 9242 was generated. Considering that cancer registries do not collect HPV status of cancers,^23^ we used CDC definitions of HPV-associated cancers with the aforementioned ICD-O-3 site codes as well as histological codes for squamous cell carcinoma (SCC) (8050-8084; 8120-8131). HPV-associated cancers were restricted to microscopically confirmed cases.^24^

We included the following demographic covariates: age at diagnosis; health insurance; county level attributes of residence (Metropolitan/Non-metropolitan, % of persons below poverty); and geographic region of the U.S. The NAACCR CiNA Research file categorizes age in 5-year intervals (e.g., 35–39 and 40–44). We grouped age into 3 categories: <54, 55–64, 65+ years. Health insurance categories included: private, Medicare, Medicaid, Other (Indian/Public Health Service, Military, TRICARE, Veterans Affairs, and insurance not otherwise specified), and no insurance or self-pay. Metropolitan/Non-metropolitan county designations were broadly based on population size (metropolitan has 50 000 persons or more). Percent of persons below poverty was categorized as: <9.99%, 10%–19.99%, and 20% or more below federal poverty levels. The 4 geographic regions of the U.S. were based on the U.S. Census Bureau: Northeast (CT, MA, ME, NH, NJ, NY, PA, RI, VT), South (AL, AR, DE, DC, FL, GA, KY, LA, MD, MS, NC, OK, SC, TN, TX, VA, WV), Midwest (IL, IN, IA, KS, MI, MN, MO, NE, ND, OH, SD, WI), and West/Pacific (AK, AZ, CO, CA, HI, ID, MT, NV, NM, OR, UT, WA, WY).

### Measures

#### Late-Stage Diagnosis

Our main outcome of interest, stage at diagnosis, was classified as local, regional, distant, or unknown, based on the SEER Summary Stage 1977/2000/Derived variable. We dichotomized stage as “early” if the tumor was identified as local disease at diagnosis and “late” stage if the tumor was regional or distant to the respective anatomic site.^25^

#### Country of origin

We used the SEER Spanish/Hispanic Origin variable in identifying the Hispanic subgroups based on country of origin. The subgroups identified by this variable included European Spanish, Cuban, Mexican, Dominican, Central/South American, and Puerto Rican. European Spanish included males identified as being born in Spain, including the Canary Islands, Balearic Islands, and Andorra.^26^ Approximately 60% of cases were classified as “Hispanic Not Otherwise Specified (NOS).” We conducted sensitivity analyses including and excluding Hispanic NOS group. Results differed; therefore, this group was included in the analysis to avoid selection bias in our results.

### Data Analysis

The number of new cases of HPV-associated cancers from 2005 to 2016 among Hispanic males was extracted from SEER*Stat and stratified by country of origin. χ^2^ tests were used to examine bivariate associations of country of origin with age, insurance status, county level attributes of residence and poverty, geographic region, stage at diagnosis, and anatomic site. Associations between demographic characteristics and late-stage diagnosis were evaluated using χ^2^ tests and univariate logistic regression. Odds ratios and corresponding 95% confidence intervals (CIs) of late-stage diagnosis compared with early stage were calculated for each category of Hispanic subgroup, with European Spanish as the reference group because preliminary analyses showed that European Spanish was similar to NH Whites (data not shown). Covariates in multivariable logistic regression models included: age at diagnosis, health insurance status, residential characteristics (i.e., metropolitan/non-metropolitan area; % poverty level of county of residence), and geographic region. Reference groups for covariates were chosen following other similar research and were generally the groups with the lowest cancer mortality.^13,15^ Lastly, interaction terms were added in the final adjusted model to examine the modification effect of Hispanic subgroup on all other variables in predicting late-stage diagnosis. No significant interactions were found (data not presented). Cases with unknown disease stage at diagnosis, health insurance status, county of residence, or unknown percent persons below poverty were excluded from the final multivariable models.

## Results

Table 1 describes our study cohort by country of origin. The largest proportion of cases were in males from Mexico (16.6%). Most of the sample consisted of Hispanic males less than 54 years of age (37.3%), with private insurance (26.4%), residing in metropolitan counties (93.5%), in counties with 10%–20% of persons below poverty (72.9%), and located in the geographic South (39.6%). Most cases were oropharyngeal cancers (67.5%) and diagnosed at regional stage of disease (55.0%).

**Table 1.** Characteristics of HPV-Associated Cancers in Hispanic Males by Country of Origin, United States, 2005–2016 (N = 9242).

Characteristic^a^	Total; N (%)	European Spanish; N (%)	Cuban; N (%)	Mexican; N (%)	Dominican; N (%)	Central/South American; N (%)	Puerto Rican; N (%)	Hispanic, NOS; N (%)
Overall	9242 (100)	321 (3.5)	425 (4.6)	1530 (16.6)	187 (2.0)	513 (5.6)	734 (7.9)	5532 (59.8)
Age at diagnosis, years								
<54	3448 (37.3)	110 (34.3)	120 (28.2)	610 (39.9)	51 (27.3)	205 (40.0)	203 (27.7)	2149 (38.8)
55-64	3037 (32.9)	97 (30.2)	140 (32.9)	476 (31.1)	64 (34.2)	177 (34.5)	272 (37.1)	1811 (32.7)
65 +	2757 (29.8)	114 (35.5)	165 (38.8)	444 (29.0)	72 (38.5)	131 (25.5)	259 (35.3)	1572 (28.4)
Residence								
Metropolitan	8643 (93.5)	292 (91.0)	414 (97.4)	1391 (90.9)	179 (95.7)	506 (98.6)	712 (97.0)	5149 (93.1)
Non-metropolitan	558 (6.0)	28 (8.7)	8 (1.9)	126 (8.2)	7 (3.7)	7 (1.4)	22 (3.0)	360 (6.5)
Insurance status								
Private	2441 (26.4)	55 (17.1)	118 (27.8)	379 (24.8)	14 (7.5)	132 (25.7)	86 (11.7)	1657 (30.0)
Medicare	2063 (22.3)	64 (19.9)	104 (24.5)	346 (22.6)	23 (12.3)	71 (13.8)	148 (20.2)	1307 (23.6)
Medicaid	1175 (12.7)	21 (6.5)	97 (22.8)	287 (18.8)	7 (3.7)	63 (12.3)	63 (8.6)	637 (11.5)
Other^b^	807 (8.7)	28 (8.7)	23 (5.4)	141 (9.2)	7 (3.7)	22 (4.3)	43 (5.9)	543 (9.8)
No insurance/Self pay	892 (9.7)	19 (5.9)	45 (10.6)	187 (12.2)	9 (4.8)	77 (15.0)	26 (3.5)	529 (9.6)
Unknown	1864 (20.2)	134 (41.7)	38 (8.9)	190 (12.4)	127 (67.9)	148 (28.8)	368 (50.1)	859 (15.5)
% persons below poverty at Residence^c^								
<9.9%	830 (9.0)	33 (10.3)	11 (2.6)	127 (8.3)	20 (10.7)	93 (18.1)	82 (11.2)	464 (8.4)
10%–19.99%	6736 (72.9)	172 (53.6)	397 (93.4)	1151 (75.2)	111 (59.4)	383 (74.7)	439 (59.8)	4083 (73.8)
> 20%	1635 (17.7)	115 (35.8)	14 (3.3)	239 (15.6)	55 (29.4)	37 (7.2)	213 (29.0)	962 (17.4)
Geographic region								
Northeast	1794 (19.4)	155 (48.3)	56 (13.2)	47 (3.1)	144 (77.0)	193 (37.6)	520 (70.8)	679 (12.3)
South	3657 (39.6)	88 (27.4)	328 (77.2)	497 (32.5)	27 (14.4)	142 (27.7)	140 (19.1)	2435 (44.0)
Midwest	605 (6.5)	6 (1.9)	9 (2.1)	96 (6.3)	9 (4.8)	18 (3.5)	26 (3.5)	441 (8.0)
West/Pacific	3186 (34.5)	72 (22.4)	32 (7.5)	890 (58.2)	7 (3.7)	160 (31.2)	48 (6.5)	1977 (35.7)
Stage at diagnosis								
Local	2193 (23.7)	69 (21.5)	73 (17.2)	329 (21.5)	39 (20.9)	134 (26.1)	157 (21.4)	1392 (25.2)
Regional	5085 (55.0)	167 (52.0)	233 (54.8)	818 (53.5)	106 (56.7)	289 (56.3)	407 (55.4)	3065 (55.4)
Distant	1461 (15.8)	69 (21.5)	85 (20.0)	277 (18.1)	34 (18.2)	71 (13.8)	132 (18.0)	793 (14.3)
Unknown	503 (5.4)	16 (5.0)	34 (8.0)	106 (6.9)	8 (4.3)	19 (3.7)	38 (5.2)	282 (5.1)
Anatomical site								
Penile	1779 (19.2)	46 (14.3)	34 (8.0)	408 (26.7)	36 (19.3)	120 (23.4)	113 (15.4)	1022 (18.5)
Anorectal	1227 (13.3)	44 (13.7)	71 (16.7)	188 (12.3)	15 (8.0)	74 (14.4)	110 (15.0)	725 (13.1)
Oropharyngeal	6236 (67.5)	231 (72.0)	320 (75.3)	934 (61.0)	136 (72.7)	319 (62.2)	511 (69.6)	3785 (68.4)

NH, Non-Hispanic.

^a^All variables were statistically significant at *P* < .01 in bivariate analyses using χ^2^ tests.

^b^Other insurance includes Indian/Public Health Service, Military, TRICARE, Veterans Affairs, and insurance not otherwise specified.

^c^Cases with unknown residence and poverty at residence are not shown due to cells having counts less than 6.

Compared with the other subgroups, a significantly higher proportion of Cubans were greater than 65 years of age (38.8%) and had Medicaid insurance (22.8%). A higher proportion of Mexicans had private insurance (27%) and lived in the Western/Pacific region of the country (59.7%). Central/South Americans composed a larger proportion of individuals with no insurance (15%) and living in counties with <9.9% poverty (18.4%). Higher proportions of oropharyngeal cancers were observed among Cubans (75.3%), Dominicans (72.7%), and European Spanish (72%), while a higher percentage of penile cancers were in Mexicans (26.7%). χ^2^ tests of these bivariate relationships were significant at *P*-values <.01.

### Late-Stage Diagnosis

#### Penile Cancers

Table 2 presents predictors of late-stage diagnosis of penile cancers among Hispanic males. Compared with European Spanish males, higher odds of late-stage diagnosis were independently observed among Mexican (adjusted odds ratio [aOR] 3.85, 95% CI 1.48–10.01) and Puerto Rican males (aOR 3.35, 95% CI 1.16–9.67). Associations were stronger after covariate adjustment. Other independent predictors of late stage diagnosis included having Medicaid (aOR 1.60, 95% CI 1.12–2.29) or no insurance (aOR 1.81, 95% CI 1.28–2.54), while age greater than 65 years was associated with lower odds (aOR .68, 95% CI 0.49–.94) of late-stage diagnosis.

**Table 2.** Predictors of Late-Stage HPV-Associated Penile Cancers Among Hispanic Males in the United States, 2005–2016 (N = 1395).

Characteristic	OR (95% CI)	aOR^a^ (95% CI)
Country of origin		
European Spanish	1.00	1.00
Cuban	1.95 (.77, 4.99)	3.19 (.99, 10.23)
Mexican	2.84 (1.46, 5.50)*	3.85 (1.48, 10.01)*
Dominican	1.75 (.69, 4.41)	1.51 (.30, 7.68)
Central or South American	1.90 (.92, 3.93)	2.53 (.91, 7.05)
Puerto Rican	2.06 (.99, 4.29)	3.35 (1.16, 9.67)*
Hispanic, NOS	1.49 (.78, 2.84)	2.38 (.93, 6.08)
Age at diagnosis (years)		
<54	1.00	1.00
55–64	1.01 (.89, 1.15)	1.21 (.91, 1.62)
65 +	.81 (.72, .90)*	.68 (.49, .94)*
Insurance type		
Private	1.00	1.00
Medicare	1.03 (.92, 1.16)	1.22 (.86, 1.74)
Medicaid	1.67 (1.38, 2.02)*	1.60 (1.12, 2.29)*
Other	.99 (.83, 1.18)	1.11 (.72, 1.71)
No insurance/Self pay	1.70 (1.40, 2.06)*	1.81 (1.28, 2.54)*
Residence		
Large metropolitan	1.00	1.00
Non-metropolitan	.92 (.83, 1.02)	1.05 (.69, 1.60)
% persons below poverty at residence		
<9.9%	1.00	1.00
10%–19.99%	.99 (.87, 1.12)	.84 (.57, 1.24)
> 20%	.92 (.79, 1.08)	.75 (.47, 1.21)
Geographic region		
Northeast	1.00	1.00
South	.92 (.81, 1.04)	1.17 (.76, 1.81)
Midwest	.98 (.85, 1.12)	1.27 (.71, 2.27)
West/Pacific	1.06 (.93, 1.21)	1.36 (.87, 2.14)

OR, odds ratio; aOR, adjusted odds ratio.

*P ≤ .01.

^a^Adjusted for all other variables in table.

#### Anorectal Cancers

Table 3 presents predictors of late-stage diagnosis of anorectal cancers among Hispanic males. Compared to European Spanish males, lower odds of late-stage diagnosis were independently observed among Central or South American (aOR .25, 95% CI 0.07–.93), Puerto Rican (aOR .23, 95% CI 0.06–.83), and Hispanic NOS males (aOR .20, 95% CI 0.06–.62). Hispanic males with Medicaid (aOR 1.98, 95% CI 1.30–3.02) or no insurance (aOR 2.19, 95% CI 1.31–3.66) had higher odds of late-stage disease at diagnosis.

**Table 3.** Predictors of Late-Stage HPV-Associated Anorectal Cancers Among Hispanic Males in the United States, 2005–2016 (N = 827).

Characteristic	OR (95% CI)	aOR^a^ (95% CI)
Country of origin		
European Spanish	1.00	1.00
Cuban	.40 (.17, .96)*	.30 (.09, 1.07)
Mexican	.49 (.23, 1.05)	.32 (.10, 1.04)
Dominican	.38 (.11, 1.33)	.33 (.03, 3.29)
Central or South American	.31 (.13, .72)*	.25 (.07, .93)*
Puerto Rican	.33 (.15, .73)*	.23 (.06, .83)*
Hispanic NOS	.25 (.12, .50)*	.20 (.06, .62)*
Age at diagnosis (years)		
<54	1.00	1.00
55–64	1.07 (.99, 1.16)	1.15 (.82, 1.62)
65 +	.90 (.83, .98)*	.87 (.58, 1.31)
Insurance type		
Private	1.00	1.00
Medicare	1.03 (.95, 1.13)	1.07 (.72, 1.60)
Medicaid	1.78 (1.57, 2.02)*	1.98 (1.30, 3.02)*^a^
Other	1.11 (.98, 1.25)	1.48 (.91, 2.40)
No insurance/Self pay	1.81 (1.56, 2.11)*	2.19 (1.31, 3.66)*
Residence		
Large metropolitan	1.00	1.00
Non-metropolitan	1.00 (.92, 1.10)	1.56 (.79, 3.07)
% persons below poverty at residence		
<9.9%	1.00	1.00
10%–19.99%	1.13 (1.02, 1.24)*	.73 (.43, 1.26)
> 20%	1.09 (.96, 1.24)	.68 (.35, 1.34)
Geographic region		
Northeast	1.00	1.00
South	1.00 (.91, 1.10)	1.35 (.81, 2.25)
Midwest	.97 (.87, 1.08)	1.08 (.54, 2.17)
West/Pacific	1.07 (.97, 1.18)	1.37 (.81, 2.31)

OR, odds ratio; aOR, adjusted odds ratio.

*P ≤ .01.

^a^Adjusted for all other variables in table.

#### Oropharyngeal Cancers

There were no differences in late-stage diagnosis of oropharyngeal cancers by Hispanic country of origin (Table 4). Similar to Hispanic males with penile or anorectal cancers, men with Medicaid or no insurance were, respectively, observed to have 50% (aOR 1.50, 95% CI 1.11–2.04) and 74% (aOR 1.74, 95% CI 1.21–2.50) higher odds of late-stage diagnosis when compared to those with private insurance, respectively. Hispanic males living in counties with 20% or more of persons below poverty were found to have lower odds of late-stage disease relative to those living in counties with <9.9% of poverty (aOR .51, 95% CI 0.34–.78).

**Table 4.** Predictors of Late-Stage HPV-Associated Oropharyngeal Cancers Among Hispanic Males in the United States, 2005–2016 (N = 4789).

Characteristic	OR (95% CI)	aOR^a^ (95% CI)
Country of origin		
European Spanish	1.00	1.00
Cuban	1.41 (.80, 2.48)	1.34 (.68, 2.64)
Mexican	1.25 (.79, 1.97)	1.17 (.65, 2.09)
Dominican	1.27 (.63, 2.56)	.65 (.24, 1.72)
Central or South American	1.17 (.68, 2.00)	.89 (.45, 1.74)
Puerto Rican	1.28 (.78, 2.12)	.98 (.50, 1.93)
Hispanic NOS	1.08 (.71, 1.63)	1.00 (.58, 1.72)
Age at diagnosis (years)		
<54	1.00	1.00
55–64	.98 (.93, 1.02)	1.01 (.81, 1.27)
65 +	.65 (.62, .68)	.75 (.57, .99)*
Insurance type		
Private	1.00	1.00
Medicare	.71 (.67, .74)*	1.12 (.85, 1.47)
Medicaid	1.45 (1.32, 1.59)*	1.50 (1.11, 2.04)*
Other	.91 (.86, .97)*	1.17 (.86, 1.59)
No insurance/Self pay	1.27 (1.14, 1.41)*	1.74 (1.21, 2.50)*
Residence		
Large metropolitan	1.00	1.00
Non-metropolitan	.89 (.85, .94)*	1.27 (.86, 1.89)
% persons below poverty at residence		
<9.9%	1.00	1.00
10%–19.99%	.93 (.88, .98)*	.85 (.59, 1.22)
> 20%	.85 (.79, .92)*	.51 (.34, .78)*
Geographic region		
Northeast	1.00	1.00
South	.78 (.74, .83)*	.76 (.53, 1.11)
Midwest	.96 (.90, 1.02)	1.03 (.62, 1.69)
West/Pacific	.96 (.90, 1.03)	1.08 (.73, 1.60)

OR, odds ratio; aOR, adjusted odds ratio.

*P ≤ .01.

^a^Adjusted for all other variables in table.

## Discussion

To the best of our knowledge, this is the first study to examine late-stage diagnosis of HPV-associated penile, anorectal, and oropharyngeal cancers among Hispanic males by country of origin. While relatively rare compared with other HPV-associated cancers, penile cancer was the only malignancy type where significant differences in stage at diagnosis by Hispanic subgroup were observed, with Mexican and Puerto Rican males having higher late-stage diagnosis compared with their European Spanish counterparts. Previous research has demonstrated that lack of circumcision and higher number of lifetime sexual partners are independent risk factors for reduced clearing of HPV and increased viral detection.^27,28^ These findings are consistent with the estimated population level circumcision rates among the country of origin subgroups included in this study, whereby Puerto Rican males have a lower proportion of male circumcision (.14%) relative to European Spanish males (6.6%).^29^ Although males from Mexico have a larger proportion of circumcision rates (15.4%), we found higher odds of late-stage diagnosis in Mexicans compared with European Spanish. This may be due to European Spanish males in the U.S. to have circumcision rates more similar to NH White males in the US (71.2%). In fact, regression modeling with NH White males as the control group found no difference in late-stage diagnosis of penile cancers with European Spanish males (aOR .98, 95% CI 0.83–1.15). This suggests that including European Spanish with other Hispanic subgroups in epidemiological cancer research may mask finding of disparities among Hispanics.

Our study also found approximately 32% lower odds of late-stage diagnosis of penile cancers among Hispanic males >65 years of age. This finding is consistent with previous research that has reported higher incidence of oncogenic and more aggressive strains of genital HPV infections among younger males relative to their older counterparts, likely due to differences in sexual risk factors.^30^

The findings from this study suggest that Central or South American as well as Puerto Rican males had lower odds of late-stage anorectal cancers compared to their European Spanish counterparts. This observation is supported by previous research reporting a higher burden of age-adjusted incidence of anal cancer in Western Europeans (.96 per 100 000 males) relative to males from the Caribbean (.18 per 100 000 males), as well as Central (.18 per 100 000 males) or South (.61 per 100 000 males) America.^31^ Additionally, HPV-associated malignancies in males has been reported to be proportionally higher in Europe than in Latin America.^32^ HPV-associated cancers of the anorectum are associated with behavioral risk factors that are more localized, such as receptive anal intercourse, higher lifetime sexual partners, prior history of sexually transmitted infections.^33^ Our findings suggest that European Spanish males may have more of these risk factors relative to Puerto Rican and Central or South American males.

Hispanic males were found to have lower odds of late-stage diagnosis of oropharyngeal cancers among individuals with >65 years of age and living in more impoverished counties. Older age and lower odds of late-stage disease is consistent with our findings for penile cancers where younger males made up a higher proportion of HPV infections with aggressive viral strains.^30^ Our observations of lower odds of late-stage diagnosis of oropharyngeal cancers in Hispanic males living in more impoverished areas can be partially explained by higher vaccine initiation and completion among males living in households with incomes below the poverty line as a result of safety-net health services.^34^ HPV-positive oropharyngeal cancers have also been reported to be more likely in younger persons, with higher SES and education, and lower exposures to tobacco and alcohol.^14,35-37^

Among all HPV-associated cancers among Hispanic men, having Medicaid and no insurance were significantly associated with higher odds of late-stage diagnosis. These results are consistent with previous research^13,15^ and probably due to lack of access to health care services as a barrier to receiving care throughout the cancer control continuum, including screening and prevention, diagnosis, treatment, survivorship, and end-of life care.^38^ Lack of insurance has been found to have a strong association with patients being diagnosed with cancer at later disease stages and having poorer survival. Similarly, existent literature identified that patients with Medicaid insurance experience advanced disease at diagnosis and worse survival.^38-41^ This may be explained by the fact that many previously uninsured patients qualify to enroll in Medicaid at or after being diagnosed with cancer, at which point available treatment modalities provide lower rates of disease eradication or control in the context of advanced disease.^38^

Research has suggested that lack of knowledge regarding HPV transmission, misperceptions about the vaccine, and lack of primary care provider (PCP) recommendation may influence parents’ decision to not vaccinate their sons.^2,11,12,42,43^ Although Hispanic parents have positive attitudes about the HPV vaccine, they were less likely to receive a recommendation from a provider and also had a slower rate of HPV vaccination uptake compared to NH Whites.^44-46^ Provider recommendation has previously been reported to be significantly associated with vaccine initiation and completion among males.^34^ Vaccination in men who have sex with men increased from 22.5% to 37.6% between 2014 and 2017 according to the American Men’s Internet Survey; however, this was only in men who had seen a PCP within the past 12 months, and had disclosed sexual preference and previous STI (both risk factors for HPV associated cancers) to their PCP.^47^ Evidence suggests geographic variations in vaccination rates, with HPV coverage being higher among adolescents living below the federal poverty level and those living in metropolitan areas. However, another study found completion of HPV vaccination was lower in low-income neighborhoods.^8,48^ It has also been reported that receipt of other vaccinations such as the meningitis vaccine was a strong indicator for likelihood of initiating and completing the HPV vaccines,^3^ which may be a potential strategy to increase HPV vaccinations among young males.

### Limitations

Although this is the first study to assess late-stage diagnosis of male HPV-associated cancers among Hispanics by country of origin, there are a few limitations to note in interpreting the results. First, this study was limited to the data within the NAACCR CiNA Deluxe file, which has a large amount of missing data pertaining to country of origin. Our analyses were conducted on cases of Hispanic males whose country of origin was known/confirmed through the NAACCR CiNA Deluxe file, and we included 12 years of data to have a sufficient number of cases for each HPV-associated cancer. We included the Hispanic NOS group in the final analyses to avoid selection bias, since this was a large proportion of cases (59.8%). Results may have differed if Hispanic country of origin were known. Second, the database also lacks details regarding other comorbidities (e.g., immunosuppression), individual behaviors (e.g., smoking status, alcohol use, and sexual activity), or other demographic characteristics (e.g., education level) that could affect stage at diagnosis for these cancers. Lastly, HPV status was not diagnostically confirmed using molecular testing for each of the cases, but instead based on the assumption that cancers from specific anatomic sites and histologies are highly associated with HPV.^23^ Therefore, our classification of HPV-associated cancers based on SCC histological type may have included cancers associated with alcohol consumption and/or smoking history, but the misclassification would be expected to be similar among the different country of origin subgroups.

## Conclusions

Mexican and Puerto Rican males had higher odds of late-stage penile cancer relative to European Spanish males, which is significant because organ preservation is less likely at late disease stage.^49^ While penile cancer remains a rare malignancy in the U.S., the physical and psychological burdens can be significant, so it is clinically important to identify populations at higher risk based on their country of origin and/or cultural practices, for example, lower rates of circumcision.^49^ Additionally, since approximately 63% of penile cancers are related to HPV^50^ and vaccinations can potentially prevent these malignancies,^51^ there is strong support to target HPV vaccinations in these specific subgroups, as well as in the larger Hispanic community. Our findings additionally suggest benefit for increasing vaccination efforts among younger Hispanic males that meet eligibility for vaccination.

When considering that 72% of oropharyngeal cancers are attributable to HPV,^50^ there is a strong suggestion that interventions to increase HPV vaccination can provide benefit among Hispanic males despite our findings not demonstrating higher odds of late-stage disease at diagnosis. Other cancer-specific health outcomes can be mitigated through vaccination particularly survival and mortality, especially when considering that Hispanics of lower SES have been previously reported to have worse outcomes relative to non-Hispanic Whites of higher SES.^16^ Our findings suggest that Hispanic males of higher SES may benefit from interventions to increase vaccination due to presenting at significantly later stages of oropharyngeal cancers relative to males of lower SES.

Because there are currently no recommended screening tests for HPV-associated cancers in males, increasing awareness of symptoms and signs of anorectal, oropharyngeal, and penile cancers are needed in the general public as well as in health care providers (e.g., primary care providers and dentists) to increase early detection of these cancers soon after presentation. Additionally, health insurance and access to health care are needed to decrease late-stage diagnosis of these preventable cancers among Hispanic males. When considering that a large proportion of the cases were categorized as Hispanic NOS within the registry, this study calls for better identification methods of Hispanic country of origin. Improved identification of these subgroups would allow for better understandings of cancer outcomes among Hispanics. Finally, more research is needed to expand the limited knowledge regarding HPV-associated cancers in this population. Inclusion of additional risk factors (e.g., sexual behaviors, circumcision, and comorbidities) and health outcomes in future research will allow greater specificity in clinical risk assessments and its implications for outreach to particular communities.
